# Transmitted Drug Resistance in Antiretroviral Therapy-Naive Persons With Acute/Early/Primary HIV Infection: A Systematic Review and Meta-Analysis

**DOI:** 10.3389/fphar.2021.718763

**Published:** 2021-11-24

**Authors:** Chunxiang Guo, Yaxin Wu, Yang Zhang, Xinchao Liu, Aixin Li, Meixia Gao, Tong Zhang, Hao Wu, Guanzhi Chen, Xiaojie Huang

**Affiliations:** ^1^ Department of Dermatology, The Affiliated Hospital of Qingdao University, Qingdao, China; ^2^ Center for Infectious Disease, Beijing Youan Hospital, Capital Medical University, Beijing, China; ^3^ Beijing Key Laboratory of HIV/AIDS Research, Beijing Youan Hospital, Capital Medical University, Beijing, China; ^4^ Department of Infectious Disease, Peking Union Medical College Hospital, Beijing, China

**Keywords:** HIV, AIDS, early infection, acute infection, primary infection, transmitted drug resistance

## Abstract

**Background:** The widespread use of antiretroviral therapy (ART) has raised concerns about the emergence of HIV transmitted drug resistance (TDR). Acute HIV infection (AHI) was the most appropriate time to detect the spread of TDR. In this meta-analysis, our purpose was to evaluate the level of TDR in ART-naive patients with primary HIV infection (PHI)/AHI/early HIV infection (EHI) and to describe the critical drug-resistant mutations.

**Methods:** We systematically searched the literature between January 1, 2008, and April 30, 2021, in PubMed, Web of Science, Embase, and the Cochrane Library. To evaluate the overall prevalence of TDR, we extracted raw data and analyzed prevalence estimates using Stata SE.

**Results:** The data of this meta-analysis come from 12 observational studies, covering 3,558 ART-naive individuals with PHI, AHI, or EHI. The overall prevalence of HIV-TDR is 9.3% (95% CI: 6.8%–11.8%, I^2^ = 81.1%, in 11 studies). The prevalence of resistance by drug class is the highest for the nonnucleoside reverse transcriptase inhibitors (NNRTIs) at 5.7% (95% CI: 2.9%–8.5%, I^2^ = 96.6%, in 11 studies), followed by nucleoside reverse transcriptase inhibitors (NRTIs) at 3.4% (95% CI: 1.8%–5.0%, I^2^ = 86.3%, in 10 studies) and protease inhibitors (PIs) at 3.3% (95% CI: 2.7%–3.9%, I^2^ = 15.6%, in 10 studies). The prevalence of TDR to integrase inhibitors (INIs) is 0.3% (95% CI: 0.1%–0.7%, I^2^ = 95.9%, in three studies), which is the lowest among all antiretroviral drugs.

**Conclusion:** The overall prevalence of TDR is at a moderate level among AHI patients who have never received ART. This emphasizes the importance of baseline drug resistance testing for public health surveillance and guiding the choice of ART. In addition, the prevalence of TDR to NNRTIs is the highest, while the TDR to INIs is the lowest. This may guide the selection of clinical antiretroviral drugs.

## 1 Introduction

With the widespread application of antiretroviral therapy (ART) globally, HIV-related morbidity and mortality have fallen dramatically (Visseaux et al., 2019). According to current guidelines, an antiretroviral regimen for a treatment-naive patients generally consists of three active agents—two nucleoside reverse transcriptase inhibitors (NRTIs) (e.g., abacavir/lamivudine) and an antiretroviral drug selected from integrase inhibitors (INIs) (e.g., dolutegravir), non-NRTI (NNRTI) (e.g., efavirenz (EFV)), or boosted protease inhibitor (PI) (e.g., lopinavir) with pharmacokinetic enhancer (World Health Organization, 2016; Department of Health and Human Services, 2019; European AIDS Clinical Society (EACS), 2019). INIs are included as part of initial HIV therapy in most treatment guidelines (Department of Health and Human Services, 2019), and NNRTI-based regimens like EFV- or rilpivirine based regimens may also be options for some patients especially in developing countries to initiate therapy (AIDS and Hepatitis C Professional Group, 2018).

But as patients have more access of ART, the risk of transmitted drug resistance (TDR) has also increased (Hamers et al., 2011; Gupta et al., 2012; Colby et al., 2016). Mistakes in HIV replication are the basis for the virus to mutate and develop drug resistance (Abram et al., 2014), which can diminish the virological response to ART (Wittkop et al., 2011). The relations between inadequate viral inhibition, poor treatment outcome, and emergence of drug resistance are well understood (Bennett et al., 2008). TDR, also known as primary drug resistance, occurs when an uninfected and ART-naive person is infected with a drug-resistant strain of HIV from someone with HIV drug-resistance mutations (DRMs). Pretreatment drug resistance (PDR) can be discovered in antiretroviral-naive people or people who start ART or had previous exposure (i.e., re-start) treatment. In summary, PDR could have been transmitted during infection (e.g., TDR), or it may be acquired after being exposed to antiretroviral drugs (e.g., acquired drug resistance), or both (Gupta et al., 2018).

TDR is a persistent public health problem that may affect ART at the population level (Bansi et al., 2010). Mathematical models also have proposed that treatment failure in patients who have not undergone ART was most likely to be caused by the preexistence of resistant mutants (Ribeiro and Bonhoeffer, 2000). It reminds us that patients with acute HIV infection (AHI) offer an occasion for real-time monitoring of TDR (Colby et al., 2016). Previous studies have shown that the prevalence of TDR during AHI is higher than that in patients with chronic HIV infections (Yanik et al., 2012). Testing for TDR during AHI can improve the sensitivity of drug-resistant strain detection (Jain et al., 2011; Yanik et al., 2012; Castro et al., 2013). Baseline testing for TDR is routinely performed in the United States and Europe (Department of Health and Human Services, 2019, European AIDS Clinical Society (EACS), 2019), but it is not the criterion in most resource-limited countries. DRMs not only severely limit the options of treatment for new patients but also accelerate the failure of treatment and thus result in a waste of medical resources. The widespread of TDR could also undermine the planning effectiveness of national treatment efforts (Wittkop et al., 2011). Therefore, routine detection of TDR is necessary to evaluate the emergence and spread of DRMs (Hedt et al., 2011; Jordan et al., 2012).

Our study mainly aims at the prevalence of TDR in AHI/early HIV infection (EHI)/primary HIV infection (PHI) patients who have not been given any antiretroviral drugs, to provide information on HIV therapy guidelines and governmental decisions on universal TDR testing, especially in some resource-limited places.

## 2 Materials and Methods

We registered our protocol with OSF (https://osf.io/evbwq/).

### 2.1 Search Strategy and Selection Criteria

We conducted this meta-analysis of the studies published during January 1, 2008, to April 30, 2021, about the prevalence of TDR in ART-naive patients with AHI/EHI/PHI, using the same search terms in PubMed, Embase, Web of Science, and the Cochrane Library (Supplementary Table S1, Supplemental Digital Content). The selection criteria of the study are as follows: Firstly, we only include original research (e.g., cohort studies, prospective studies, case–control studies, and randomized controlled trials) on adults (aged >15 years), excluding review articles and non-English studies. Secondly, the inclusion criteria only considered initial studies whose experiments must be done after January 1, 2000. We excluded studies of patients who had been exposed to antiretroviral drugs or those who were not in the acute phase of HIV infection. We excluded those studies that do not describe detailed information on drug resistance and patients’ treatment involving ART, as well as the ones that focus on patients with mother-to-child transmission and coinfection. We also excluded studies that were mixed with recent or chronic infections or unable to provide objective data.

### 2.2 Study Selection and Extraction

Two investigators (CG and YW) independently screened the titles and summaries, then evaluated the full text of the record, and extracted the relevant data. Divergences were settled through consensus or a third reviewer’s (YZ’s) arbitration.

We extracted the following data from each study: country; sex; age; risk groups; study type; year of sample collection; number of participants; and the number of patients with more than one DRMs, with one or more NRTI mutation, with one or more NNRTI mutation, with one or more PI mutation, with one or more INI mutation, and with one or more Fusion enzyme inhibitors and their respective mutation sites.

### 2.3 Risk of Bias Assessment

We used the Newcastle–Ottawa Quality Assessment Scale (NOS) for the non-randomized trial and the observational study to evaluate the bias quality and risk of the included studies, including the study population, the comparability between groups, and the measurement of results (Wells et al., 2014). The more the number of stars (☆), the better the quality is, and studies rated five stars or more can be included in the analysis. A funnel plot and Egger’s test were applied to detect any publication bias. A *p*-value of <0.05 was considered statistically significant.

### 2.4 Data Analysis

The main purpose of this meta-analysis is to describe the prevalence of TDR to NRTIs, NNRTIs, PIs, and INIs in ART-naive patients with AHI, EHI, or PHI.

As for HIV genotype sequences, we focused on the mutations sites in Stanford University Drug Resistance Database and the WHO surveillance drug resistance mutation list (Bennett et al., 2009; Stanford, 2020). The WHO classifies the level of TDR as low (<5%), moderate (5%–15%), and high (>15%) (Jordan et al., 2012).

Statistical analysis was done in Stata SE. Heterogeneity was assessed using the I^2^ statistic; when I^2^ <25%, 25%–50%, and >50%, the heterogeneity was low, medium, and high, respectively. If I^2^ is lower than 50%, we apply the fixed model; otherwise, the random model. Subgroup analysis was conducted according to study country to assess the heterogeneity between studies. We set the confidence interval as 95% for each estimate. We calculated the proportion of specific mutants after crudely pooling the numbers of individuals with any mutation and the number with particular mutations (Gupta et al., 2018).

## 3 Results

### 3.1 Study Included and Characteristics

**FIGURE 1:**
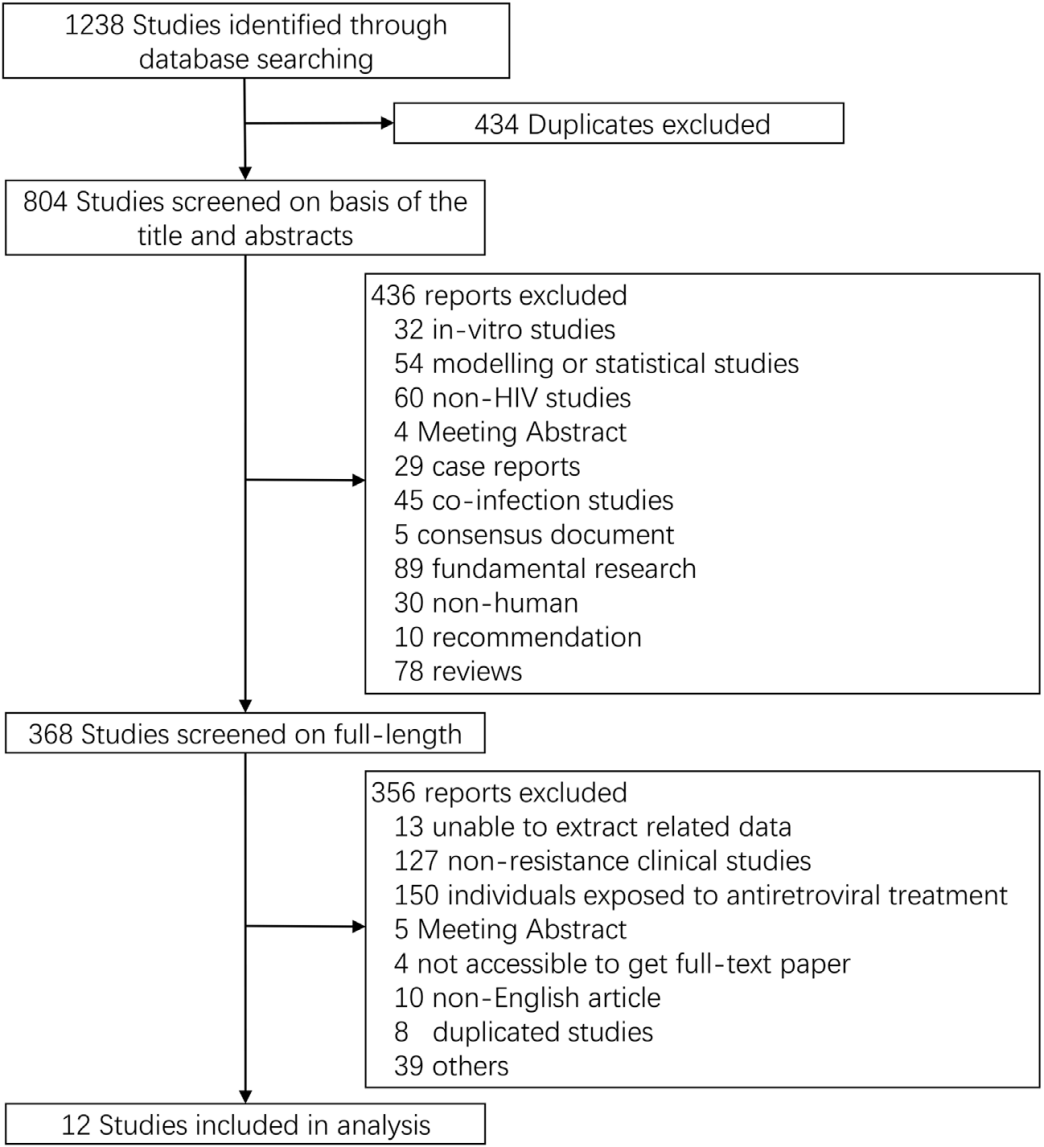
Flow diagram of search results and screening process.

We initially identified 1,238 potential records from four electronic databases according to our search strategy. After deleting studies that do not meet our inclusion criteria, 12 full-length papers were analyzed, which represented 3,558 participants with data on TDR-associated mutations (TDRAMs) (Figure 1) (Kim et al., 2008; Castor et al., 2012; Yanik et al., 2012; Dai et al., 2014; Ambrosioni et al., 2015; Ananworanich et al., 2015; Stekler et al., 2015; Zhao et al., 2015; Colby et al., 2016; Panichsillapakit et al., 2016; Rutstein et al., 2019; Visseaux et al., 2019). One of the literatures included in this study was a cross-sectional survey, and 11 were cohort study. The sampling span was from 2000 to 2016, and there were eight studies with a sample size of more than 100. Most of the study data came from voluntary counseling and testing institutions, blood donation centers, hospitals, and pathology laboratories. Four of the seven countries involved in the study are developed countries. The characteristics of concerning studies are listed in Table 1.

**TABLE 1:** Characteristics of studies included in the meta-analysis.

Author, publication year, references	Country	Age (years)		Sample size	HIV risk groups	Year of sample collection	Study design (source of data)
		Range	Median (IQR) or mean ± SD				
Kim et al. (2008)	Korea	15–75	—	66	48.5% HTS	2002.03–2005.06	Retrospective cohort study (historic medical records)
					46.9% homosexual contact		
					4.6% Unknown		
Castor et al. (2012)	The United States	15–69	—	497	—	2000–2010.12	Retrospective study (historic medical records)
Yanik et al. (2012)	The United States	≥18	—	43	81% MSM	2000–2010.9	Retrospective cohort study (historic medical and lab records)
					19% Unknown		
Dai et al. (2014)	China	—	31.8 ± 8.7	266	MSM	2008–2011	Prospective cohort study (medical records)
Ambrosioni et al. (2015)	Spain	—	33 (28–39)	161	82% MSM, 11.8% HTS, 3.1% IDUs, 3.1% unknown	2000–2012	Retrospective cohort study (historic medical records)
Ananworanich et al. (2015)	Thailand	—	28 (24–32)	120	90% MSM, 10% HTS	2009.04.20–2013.09.13	Prospective cohort study (clinic records)
Zhao et al. (2015)	China	18–70	—	367	MSM	2012–2013	Cross-sectional survey (crowd serologic screening)
Stekler et al. (2015)	The United States	—	31 (25–41)	82	MSM	2007–2013	Retrospective cohort study (historic medical records)
Colby et al. (2016)	Thailand	—	28 (23–32)	233	93% MSM, 7% HTS	2009.04.20–2014.12.31	Prospective cohort study (HIV testing centers)
Panichsillapakit et al. (2016)	The United States	>18	—	456	—	2000–2013	Retrospective cohort study (San Diego Primary Infection Cohort)
Rutstein et al. (2019)	Malawi	>18	—	45	—	2012.06–2014.01	Retrospective cohort study (re-analyzing lab samples)
Visseaux et al. (2019)	France	—	36 (28–45)	1,121	70% MSM, 18% HTS, 12% unknown	2014–2016	Retrospective cohort study (re-analyzing lab samples)

Note: MSM, men who have sex with men; HTS, heterosexuals; IDUs, injection drug users; IQR, interquartile range; SD, standard deviation.

### 3.2 The Prevalence of Transmitted Drug Resistance and Transmitted Drug Resistance-Associated Mutations

#### 3.2.1 The Overall Prevalence of Transmitted Drug Resistance

**FIGURE 2:**
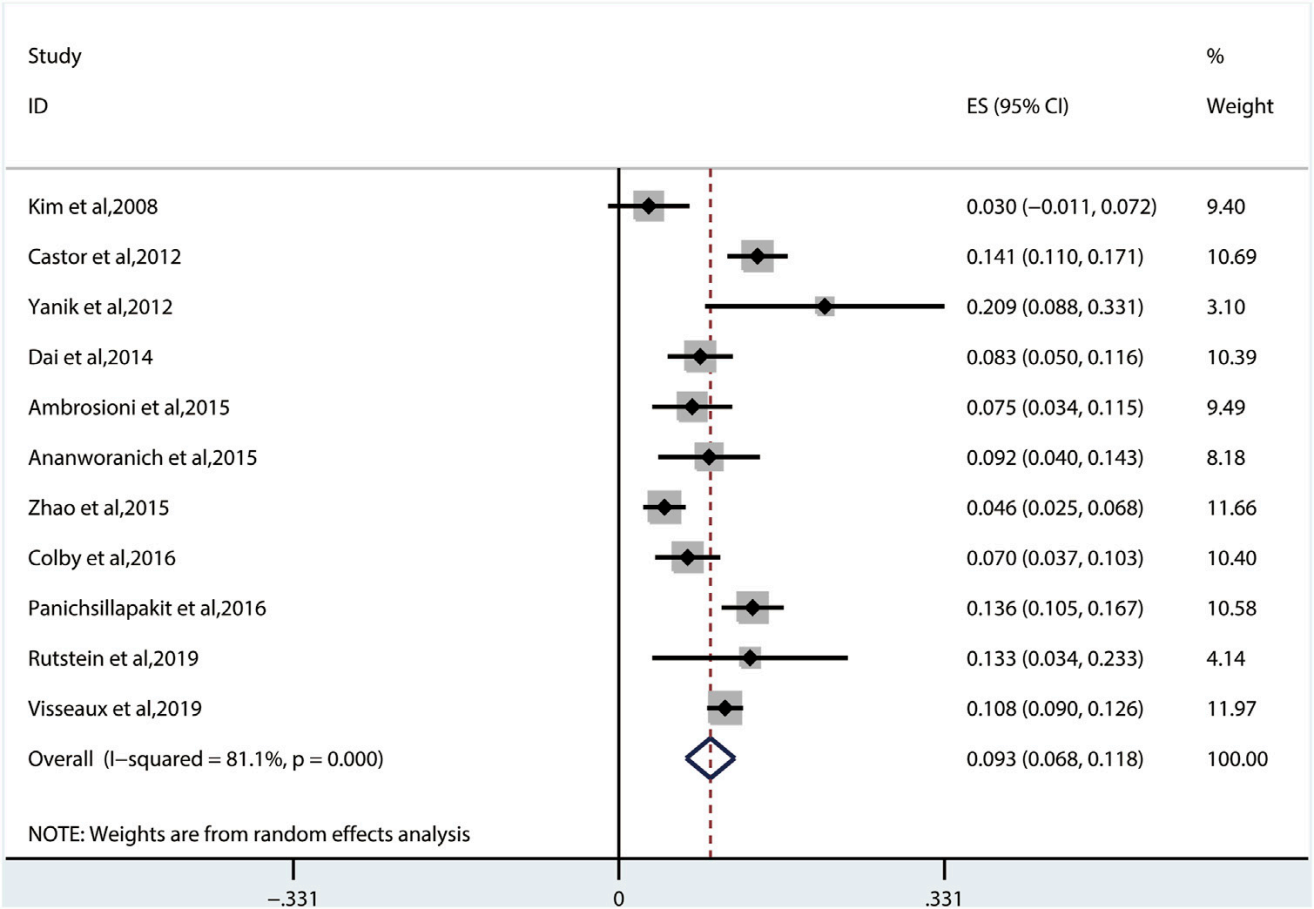
The total prevalence of TDR. Note: the red dotted line indicates the overall prevalence of TDR. TDR, transmitted drug resistance.

**FIGURE 3:**
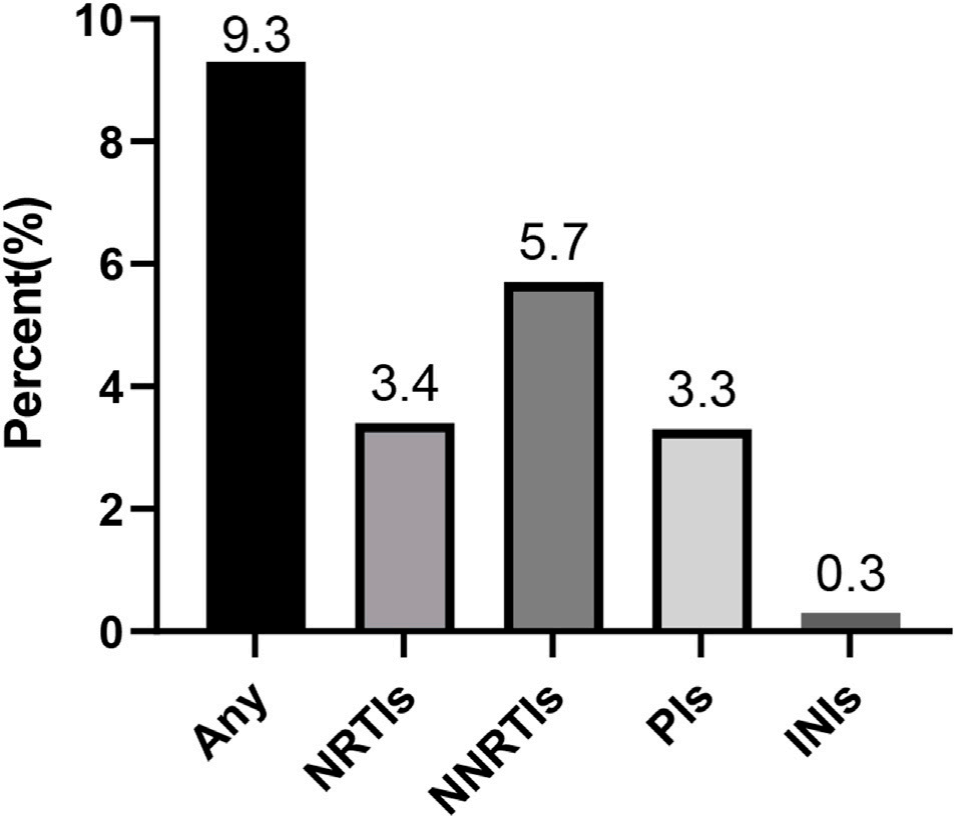
Presentation of the prevalence of TDR. Note: NRTIs, nucleoside reverse transcriptase inhibitors; NNRTIs, nonnucleoside reverse transcriptase inhibitors; PIs, protease inhibitors; INIs, integrase inhibitors; TDR, transmitted drug resistance.

The random-effects model to obtain the overall prevalence of TDR is 9.3% (95% CI: 6.8%–11.8%, I^2^ = 81.1%) among 11 studies (Figure 2) (Kim et al., 2008; Castor et al., 2012; Yanik et al., 2012; Dai et al., 2014; Ambrosioni et al., 2015; Ananworanich et al., 2015; Zhao et al., 2015; Colby et al., 2016; Panichsillapakit et al 2016; Panichsillapakit et al., 2016; Rutstein et al., 2019; Visseaux et al., 2019). Subgroup analysis shows that the prevalence of TDR in developed and developing countries is 10.9% (95% CI: 9.6%–12.1%, I^2^ = 0%, in six studies) and 6.4% (95% CI: 4.9%–7.9%, I^2^ = 0%, in five studies), respectively (Supplementary Figure S1). Overall TDR prevalence and NRTI-, NNRTI-, PI-, and INI-related TDR are shown in Figure 3, which is analyzed by different underlying studies. There were no data of TDR to Fusion enzyme inhibitors. Publication bias, as examined by the funnel plot (Supplementary Figure S2) and Egger’s test (*p* = 0.673), showed no publication bias in this analysis.

#### 3.2.2 Transmitted Drug Resistance of Nucleoside Reverse Transcriptase Inhibitors

**FIGURE 4:**
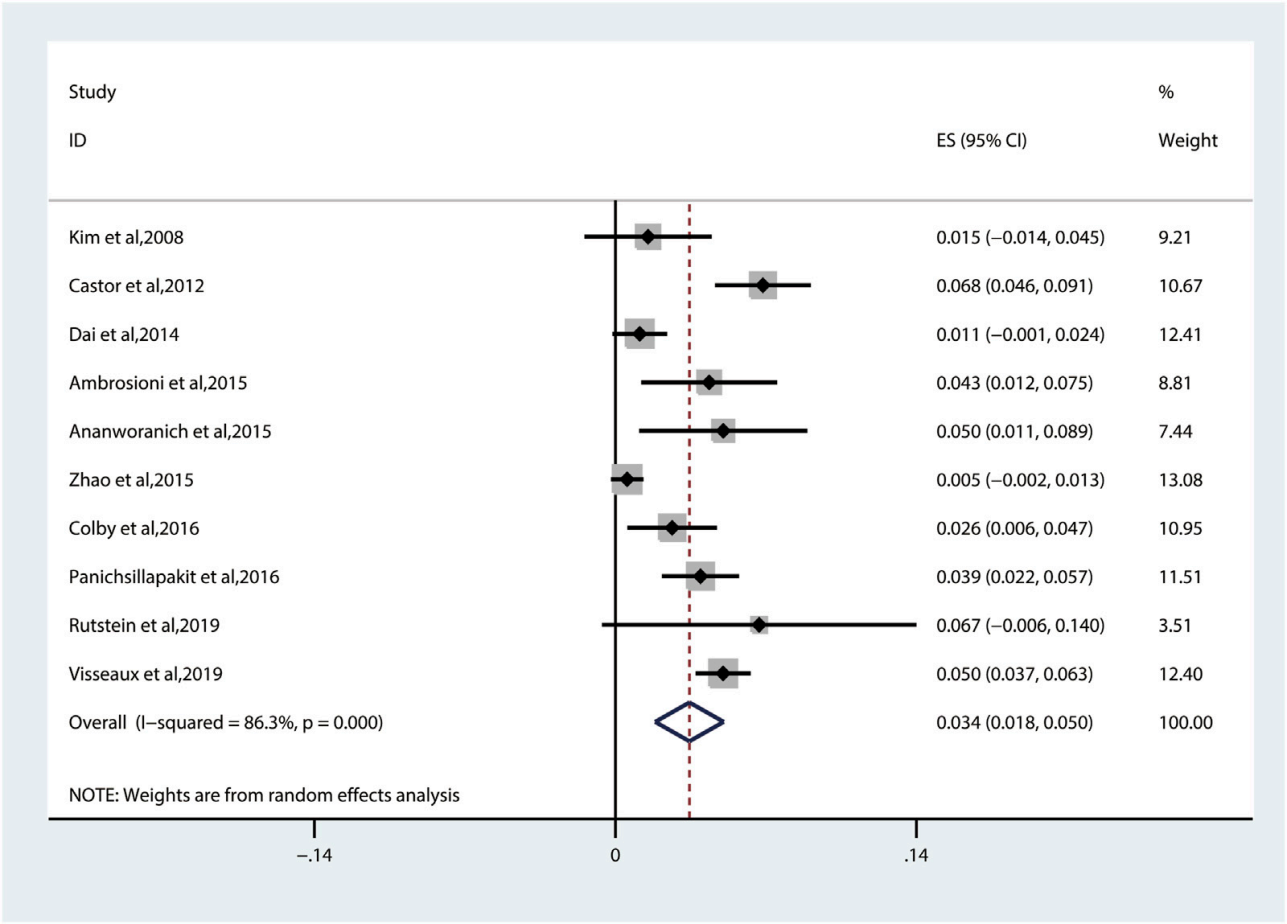
The prevalence of TDR to NRTIs. TDR, transmitted drug resistance; NRTIs, nucleoside reverse transcriptase inhibitors.

**FIGURE 5:**
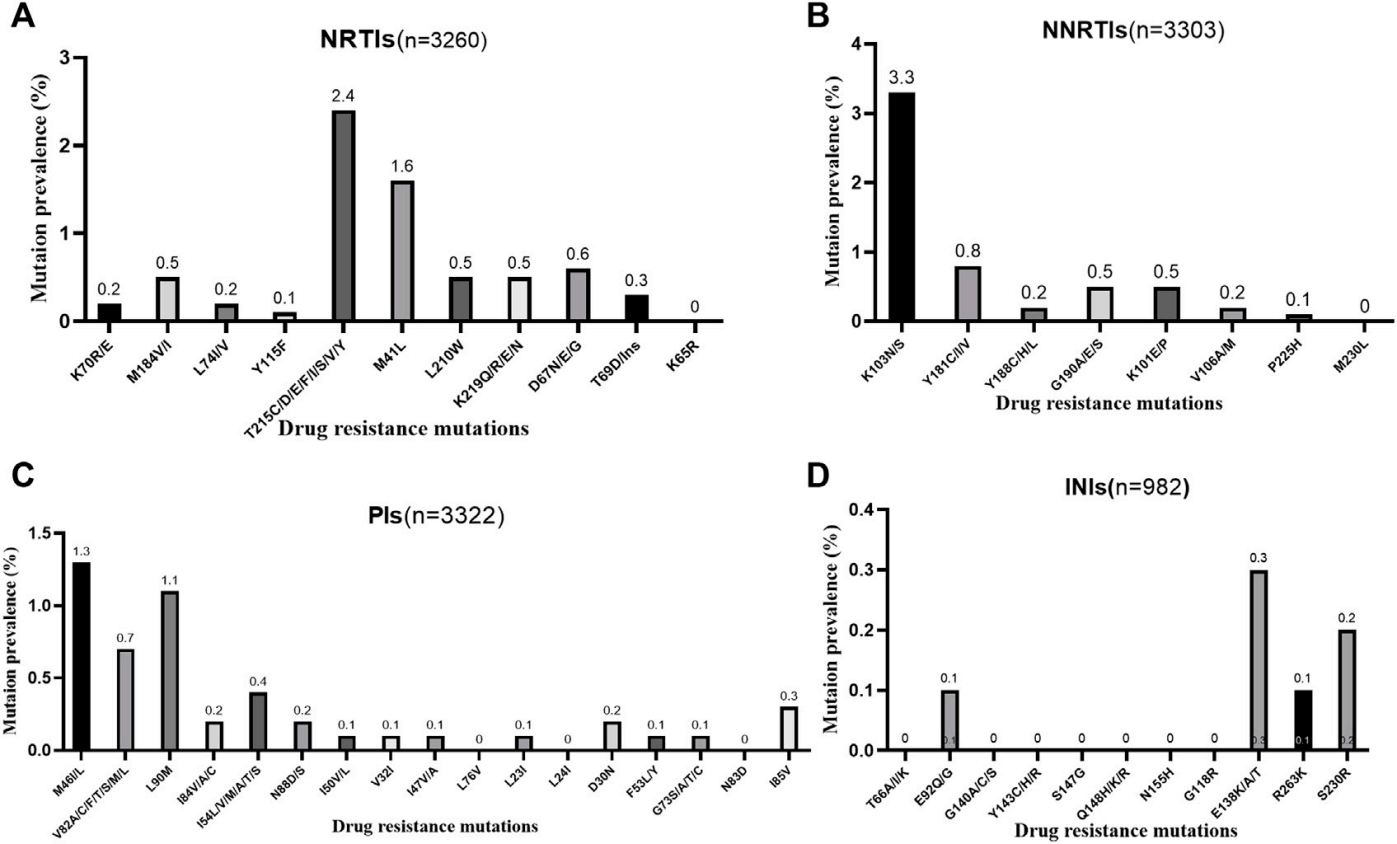
Crude prevalence of drug‐resistance mutations in people with any mutation. Note: **A,** NRTI-associated drug-resistance mutations. **B,** NRTI-associated drug-resistance mutations. **C,** PI-associated drug-resistance mutations. **D,** INI-associated drug-resistance mutations. NRTIs, nucleoside reverse transcriptase inhibitors; NNRTIs, non-nucleoside reverse transcriptase inhibitors; PIs, protease inhibitors; INIs, integrase inhibitors.

The pooled TDRAMs of NRTIs are 3.4% (95% CI: 1.8%–5.0%, I^2^ = 86.3%) (Figure 4) reported in 10 studies (Kim et al., 2008; Castor et al., 2012; Dai et al., 2014; Ambrosioni et al., 2015; Ananworanich et al., 2015; Zhao et al., 2015; Colby et al., 2016; Panichsillapakit et al., 2016; Rutstein et al., 2019; Visseaux et al., 2019). Among 3,260 patients, the most common TDRAMs for the NRTIs are T215C/D/E/F/I/S/V/Y mutations (2.4% patients), followed by M41L (1.6%), which mainly produces resistance to zidovudine and stavudine. Rare TDRAMs about NRTI-associated position are T69D/Ins (0.3%), K70R/E (0.2%), L74I/V (0.2%), and Y115F (0.1%), especially K65R (0%), which is only found in one patient (Figure 5A).

#### 3.2.3 Transmitted Drug Resistance of Nonnucleoside Reverse Transcriptase Inhibitors

**FIGURE 6:**
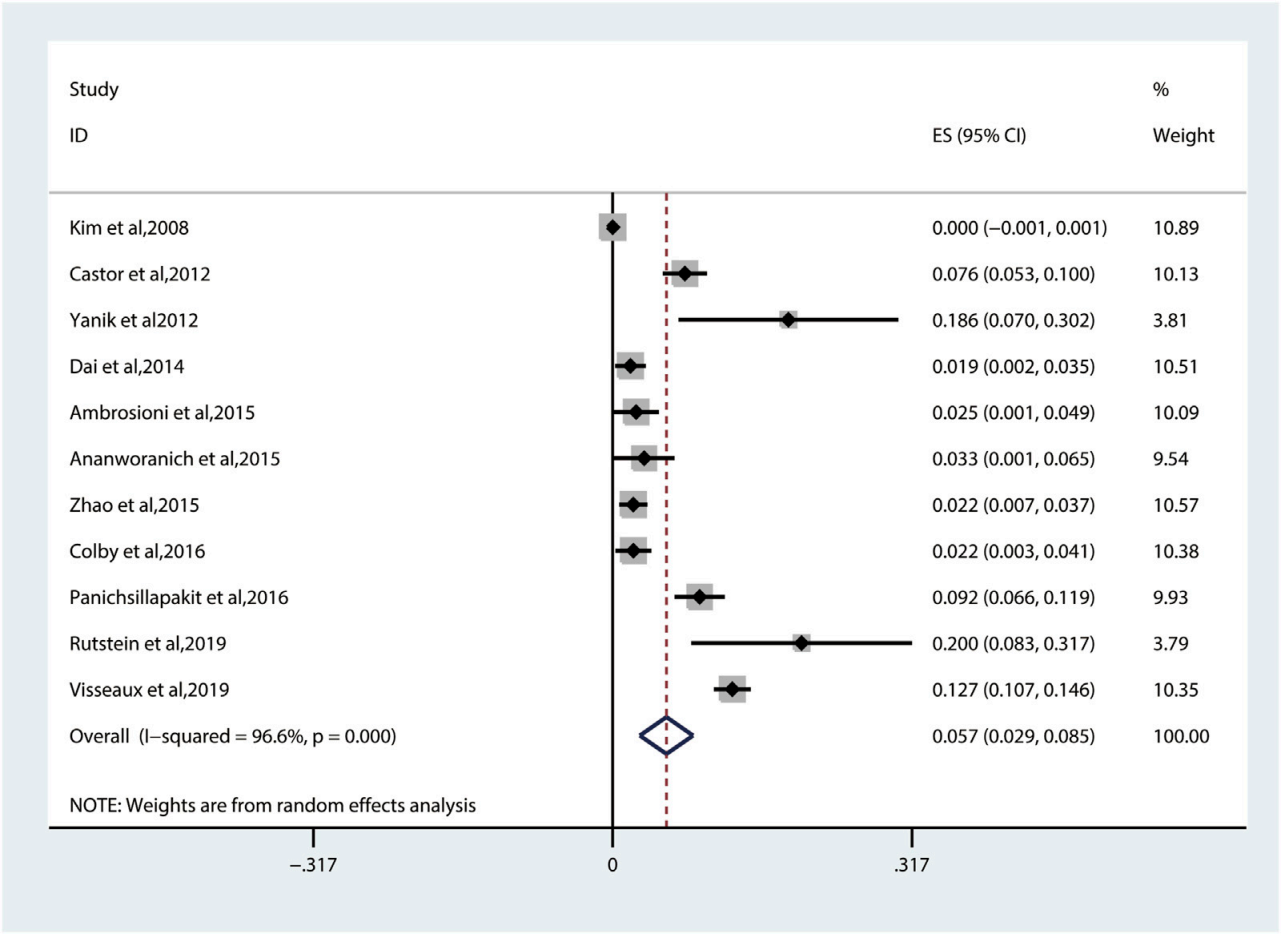
The prevalence of TDR to NNRTIs. TDR, transmitted drug resistance; NNRTIs, nonnucleoside reverse transcriptase inhibitors.

The prevalence of TDR to NNRTIs (5.7%, 95% CI: 2.9%–8.5%, I^2^ = 96.6%, reported in 11 studies) (Figure 6) is the highest among all kinds of inhibitors (Figure 3) (Kim et al., 2008; Castor et al., 2012; Yanik et al., 2012; Dai et al., 2014; Ambrosioni et al., 2015; Ananworanich et al., 2015; Zhao et al., 2015; Colby et al., 2016; Panichsillapakit et al., 2016; Rutstein et al., 2019; Visseaux et al., 2019). Among these 11 studies, 10 studies have descriptions of TDRAMs about NNRTIs, in which they detect eight mutations in 3,303 patients. The most common NNRTI-related genotypic mutations are K103N/S (3.3% patients), which develop resistance to EFV or nevirapine (NVP), followed by Y181C/I/V (0.8% patients). Among eight TDRAMs, P225H (0.1% patients) and M230L (0% patients) are relatively uncommon, each of which was found in less than four patients (Figure 5B).

#### 3.2.4 Transmitted Drug Resistance of Protease Inhibitors

**FIGURE 7:**
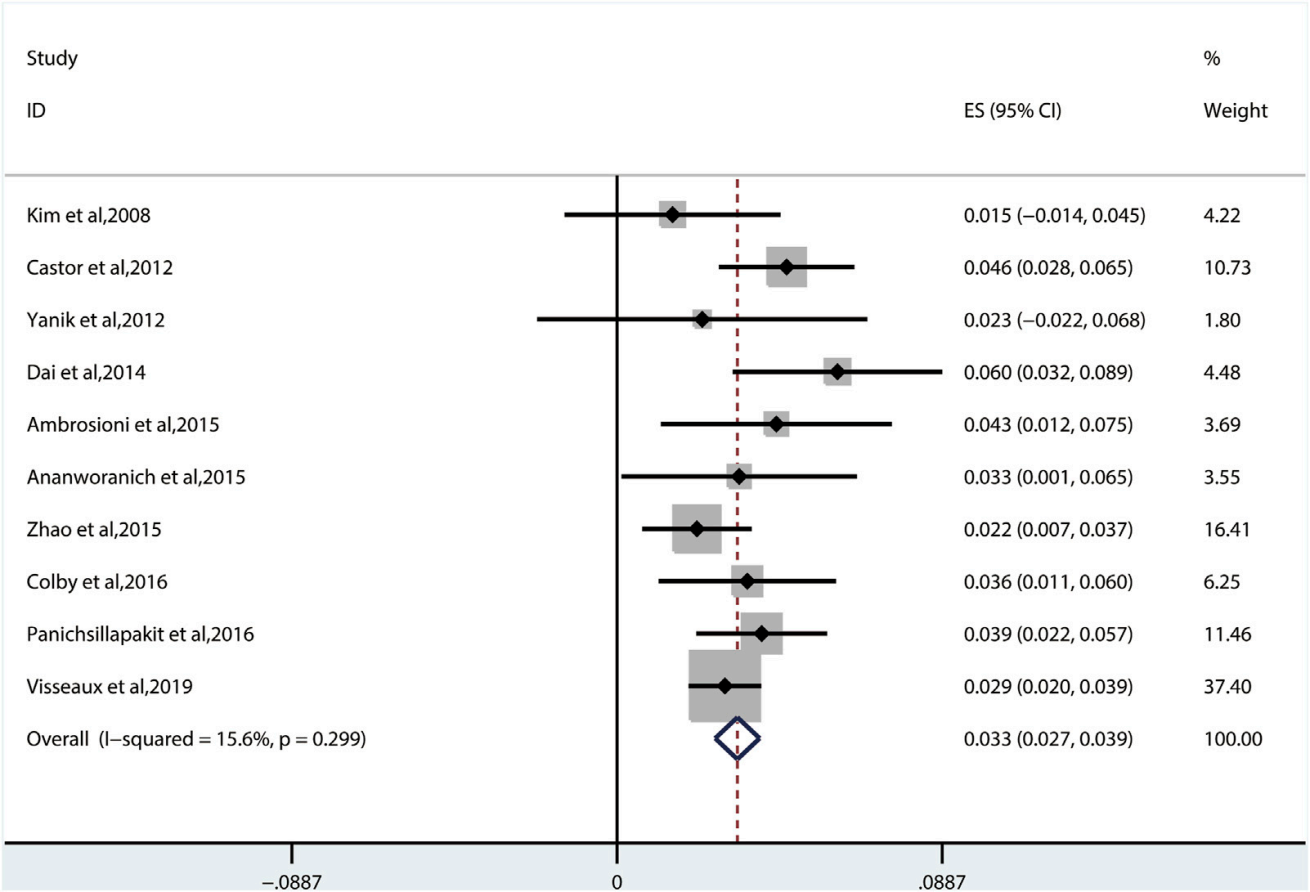
The prevalence of TDR to PIs. TDR, transmitted drug resistance; PIs, protease inhibitors.

We used the fixed-effects model to obtain the prevalence of TDR to PIs of 3.3% (95% CI: 2.7%–3.9%, I^2^ = 15.6%, reported in 10 studies) (Figure 7), which is lower than that to NNRTIs and NRTIs (Kim et al., 2008; Castor et al., 2012; Yanik et al., 2012; Dai et al., 2014; Ambrosioni et al., 2015; Ananworanich et al., 2015; Zhao et al., 2015; Colby et al., 2016; Panichsillapakit et al., 2016; Visseaux et al., 2019). Among all the patients harboring PI-associated resistant mutations detected in 10 studies, the most common TDRAMs are M46I/L mutations (found in 44 patients), which reduces susceptibility to nelfinavir (NFV). Besides, 57 people had PI-related major genotypic mutation at L90M (1.1% patients) and V82A/C/F/T/S/M/L (0.7% patients). Rare TDR mutations about PI-related minor genotypic mutations are L76V, N83D, and L24I mutations, which are found in one person each (Figure 5C).

#### 3.2.5 Transmitted Drug Resistance of Integrase Inhibitors

**FIGURE 8:**
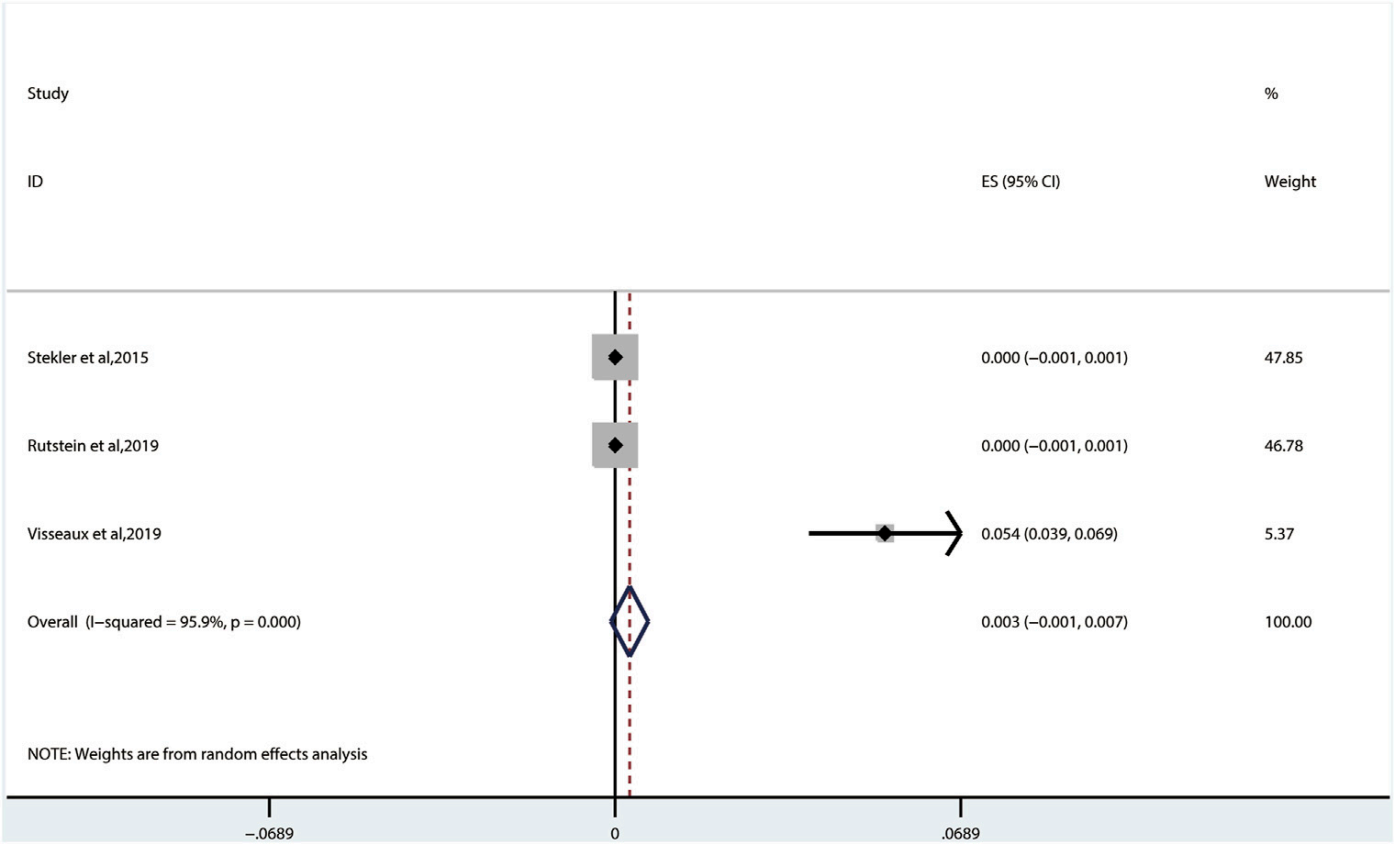
The prevalence of TDR to INIs. TDR, transmitted drug resistance; INIs, integrase inhibitors.

The prevalence of TDRAMs to INIs (0.3%, 95% CI: −0.1%–0.7%, I^2^ = 95.9%, in three studies) (Figure 8) is the lowest in ART-naive persons with AHI/EHI/PHI (Figure 3) (Stekler et al., 2015; Rutstein et al., 2019; Visseaux et al., 2019). There are no INI mutations detected in two studies (Stekler et al., 2015; Rutstein et al., 2019). One of them identified the viral polymorphisms in INIs in 24 subjects, including V54I (*n* = 1), L74I (*n* = 7), V151I (*n* = 3), M154I/L (*n* = 4), G163E (*n* = 1), I203M (*n* = 2), and S230N (*n* = 6), and identified no subjects with major INI mutations (Stekler et al., 2015). E92Q/G (0.1% patients), E138K/A/T (0.3% patients), and R263K (0.1% patients) in major INI mutations are found in only one study (Figure 5D) (Visseaux et al., 2019).

### 3.3 Quality Assessment

The NOS is used to evaluate the literature quality and bias risk of non-randomized controlled trials (Wells et al., 2014). The quality evaluation of the 12 included studies is all above five stars (Supplementary Table S2). Participation bias may result from particular participant eligibility criteria in most studies. Participant retention was high overall. As shown earlier, generally speaking, no significant publication bias was shown.

## 4 Discussion

We observed an overall TDR prevalence of 9.3% (95% CI: 6.8%–11.8%) in this meta-analysis. Subgroup analysis shows that the prevalence of TDR in developed countries is higher than that in developing countries, and both at a moderate level (Jordan et al., 2012). Among different categories of HIV drugs, patients show the highest resistance to NNRTIs, while they show the lowest to the TDRAMs in INIs. This will help inform donors and policymakers on the urgent need to address drug resistance in AHI/EHI/PHI people without ART in an environment where high epidemic resistance has emerged.

At present, the antiretroviral regimen for treatment-naive patients generally consists of two NRTIs plus a third active agent selected from INIs, NNRTIs, or boosted PIs with pharmacokinetic enhancers (Department of Health and Human Services, 2019; European AIDS Clinical Society (EACS), 2019; World Health Organization, 2016). NRTIs play a central role in ART and are widely used in the treatment of HIV infection. In the era of pre-combined ART, TDR was mainly targeted at NRTIs (Panichsillapakit et al., 2016). In our analysis, the TDR prevalence to NRTIs was second to that to NNRTIs. NRTI-related mutations, especially those that reduce the fitness of the virus, can gradually disappear (such as M184V) (Ambrosioni et al., 2015). Since NNRTIs were widely used, the prevalence of TDR to NNRTIs has increased over time (Panichsillapakit et al., 2016). Our analysis indicates that the TDR of NNRTIs was the highest among the four types of inhibitors. And the most common NNRTI-associated mutations are K103N/S, which can largely reduce the susceptibility or virological response and develop resistance to NVP or EFV (Stanford, 2020). A single amino acid mutation can produce resistance to NVP or EFV, and the DRMs in NNRTIs are particularly important in predicting the effectiveness of first-line regimens (Zhao et al., 2015; Avila-Rios et al., 2016). Besides, a few variants may persist and have a negative impact on the response to treatments (Jain et al., 2011; Li et al., 2011). But in some developing countries, NNRTI-based (e.g., EFV- or rilpivirine-based) programs are still the choice for some persons infected with HIV to begin their treatments (AIDS and Hepatitis C Professional Group, 2018). The TDR prevalence to PIs was lower than that to NNRTIs and NRTIs, and the clinical consequences caused by the increase of single PI resistance mutations are very limited because of the high genetic barrier (Rhee et al., 2010; Wensing et al., 2010; Ambrosioni et al., 2015). Nowadays, the two-drug regimens containing dolutegravir + lamivudine have become a relatively new scheme recommended as first-line ART (Department of Health and Human Services, 2019). The existing data have shown that the TDR of INIs was the lowest in all kinds of inhibitors. Since 2014, INIs were listed as the preferred option for ART-naive patients due to their excellent efficacy and safety (Walmsley et al., 2013; Clotet et al., 2014; Lennox et al., 2014; Squires et al., 2016). It is worth mentioning that although cases of drug resistance transmission of INIs have been reported, major INI mutations are rarely found (Stekler et al., 2015; Rutstein et al., 2019). By this token, a PI-based and/or an INI-based combination over NNRTI-based regimens should reduce the risk of early failure (Visseaux et al., 2019). At the same time, as more and more patients choose INI-based regimens, we should also be prepared for a possible increase in the spread of INI mutations (Stekler et al., 2015).

The emergence of HIV drug resistance may impair the effectiveness of antiretroviral drugs and further impact global HIV response and ART promotion (Zhao et al., 2015; Clutter et al., 2016). According to current guidelines, drug resistance tests were recommended in all cases right after the diagnosis is made (Department of Health and Human Services, 2019; European AIDS Clinical Society (EACS), 2019). Detection of DRMs in AHI patients may be an important means to predict future drug resistance patterns and help provide information on both global guidelines for the treatment and management of HIV (Yanik et al., 2012).

As far as we know, this is the first meta-analysis of TDR and mutations in ART-naive person with PHI, AHI, or EHI. We applied a systematic, comprehensive, and quantitative method in the analysis and collected relatively comprehensive data and results of DRMs in the acute phase of HIV infection.

There are also limitations in our study. Firstly, similar to most meta-analyses, the analyzed data were extracted from the literature, rather than the original data, which may lead to a small bias in data selection. Secondly, because there were relatively few tests for DRMs in ART-naive patients with PHI, AHI, or EHI, the analysis only includes a small number of studies. Thirdly, the data used in the study were mostly from urban and peri-urban areas, so the analysis mainly reflects the situation of DRMs in these areas. Despite these limitations, our meta-analysis can still provide references for ART-naive patients with AHI to choose antiretroviral regimens before starting treatment, especially in countries with NNRTI-based ones.

## 5 Conclusion

The moderate TDR prevalence detected among ART-naive patients with AHI/EHI/PHI in this systematic review and meta-analysis highlights the significance of routine drug resistance detection before starting ART in both developed and developing countries. The finding that a few NNRTI-associated (K103N/S and Y181C/I/V) DRMs were responsible for most cases of TDR suggests that the current available first-line ART regimens containing EFV or NVP should be urgently amended. On the basis of these findings, the direction of our follow-up works lies in expanding a variety of viral load measurements and further optimizing ART regimens.

## Supplementary Material

Supplementary Figure 1 Result of subgroup analysis based on study country.

Supplementary Figure 2 Funnel plot with pseudo 95% CI in this analysis.

## Abbreviations

HIV, human immunodeficiency virus; AIDS, acquired immuno deficiency syndrome; ART, antiretroviral therapy; AHI, acute HIV infection; PHI, primary HIV infection; EHI, early HIV infection; TDR, transmitted drug resistance; WHO, world Health organization; TDRAMs, transmitted drug resistance-associated mutations; DRMs, drug-resistance mutations; NRTIs, nucleoside reverse transcriptase inhibitors; NNRTIs, nonnucleoside reverse transcriptase inhibitors; INIs, integrase inhibitors; EFV, efavirenz; NVP, nevirapine.
