# Supplementary figures and images for: Transmitted Drug Resistance in Antiretroviral Therapy-Naive Persons With Acute/Early/Primary HIV Infection: A Systematic Review and Meta-Analysis
Source: Front Pharmacol. 2021 Nov 24;12:718763. doi: 10.3389/fphar.2021.718763 (PMC8652085; doi:10.3389/fphar.2021.718763)

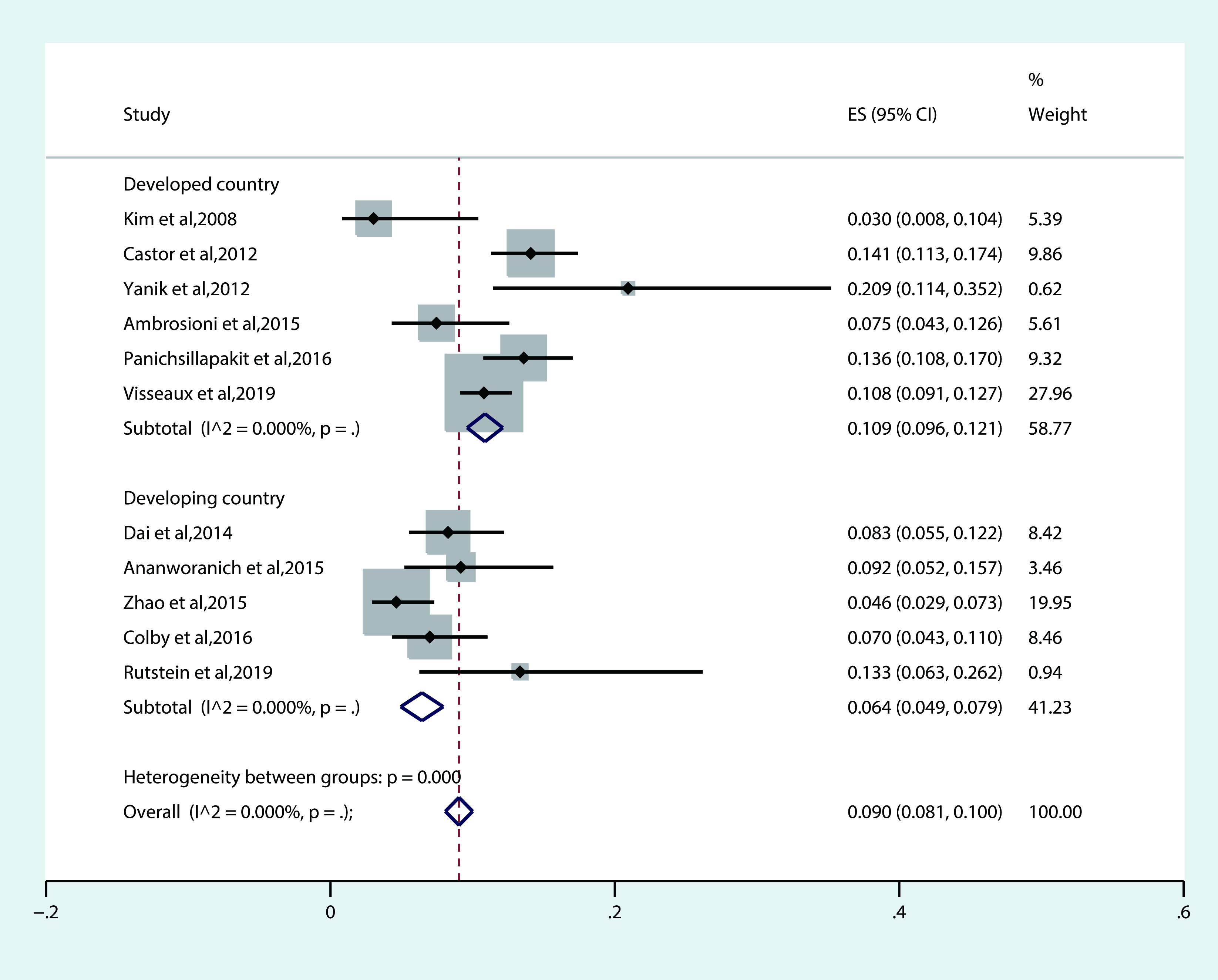

Supplement: Supplementary file 1 [file Image1.JPEG]

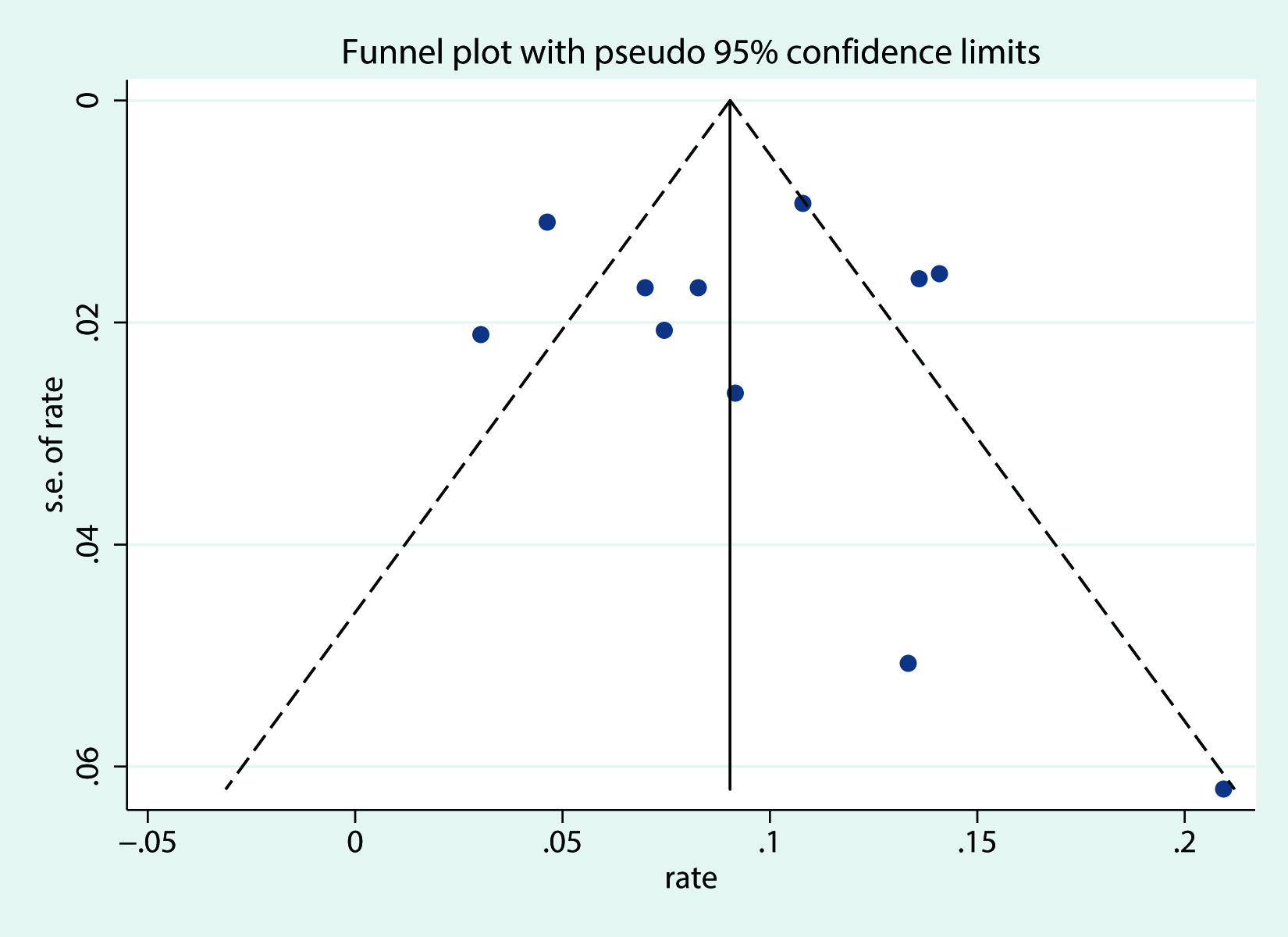

Supplement: Supplementary file 2 [file Image2.JPEG]
