# Supplementary material for: Transmitted Drug Resistance in Antiretroviral Therapy-Naive Persons With Acute/Early/Primary HIV Infection: A Systematic Review and Meta-Analysis
Source: Front Pharmacol. 2021 Nov 24;12:718763. doi: 10.3389/fphar.2021.718763 (PMC8652085; doi:10.3389/fphar.2021.718763)
Supplement: Supplementary file 3 [file Table1.DOC]

**Table S1.** Search strategies for PubMed.

| **Term group** | **Query** | **Search Terms** | **Tags** |
| --- | --- | --- | --- |
| HIV/AIDS terms | #1 | HIV Infection | MESH |
|  | #2 | HIV | MESH |
|  | #3 | HIV | TIAB |
|  | #4 | Human Immunodeficiency Virus | TIAB |
|  | #5 | Human Immunedeficiency Virus | TIAB |
|  | #6 | Human Immuno-deficiency Virus | TIAB |
|  | #7 | Human Immune-deficiency Virus | TIAB |
|  | #8 | Acquired Immunodeficiency Syndrome | TIAB |
|  | #9 | Acquired Immunedeficiency Syndrome | TIAB |
|  | #10 | Acquired Immuno deficiency Syndrome | TIAB |
|  | #11 | Acquired Immune deficiency Syndrome | TIAB |
|  | #12 | AIDS | TIAB |
|  | #13 | #1 OR #2 OR #3 OR #4 OR #5 OR #6 OR #7 OR #8 OR #9 OR #10 OR #11 OR #12 | |
| Infection terms | #14 | AHI | MESH |
|  | #15 | AHI | All Fields |
|  | #16 | Acute HIV infection | All Fields |
|  | #17 | Acute infection | All Fields |
|  | #18 | EHI | All Fields |
|  | #19 | Early HIV infection | All Fields |
|  | #20 | Early infection | All Fields |
|  | #21 | PHI | All Fields |
|  | #22 | Primary HIV infection | All Fields |
|  | #23 | Primary infection | All Fields |
|  | #24 | #14 OR #15 OR #16 OR #17 OR #18 OR #19 OR #20 OR #21 OR #22 OR #23 | |
| Resistance terms | #25 | Resistance | All Fields |
|  | #26 | TDR | All Fields |
|  | #27 | Transmitted drug resistance | All Fields |
|  | #28 | Primary drug resistance | All Fields |
|  | #29 | PDR | All Fields |
|  | #30 | Pretreatment drug resistance | All Fields |
|  | #31 | #25 OR #26 OR #27 OR #28 OR #29 OR #30 | |
| Published time | #32 | 2008/01/01 to 2021/04/30 | Date - Publication |
| **Complete search** | #33 | #13 AND #24 AND #31 AND #32 | |
