# Supplementary material for: Transmitted Drug Resistance in Antiretroviral Therapy-Naive Persons With Acute/Early/Primary HIV Infection: A Systematic Review and Meta-Analysis
Source: Front Pharmacol. 2021 Nov 24;12:718763. doi: 10.3389/fphar.2021.718763 (PMC8652085; doi:10.3389/fphar.2021.718763)
Supplement: Supplementary file 4 [file Table2.DOC]

**Table** **S2**: Summary of appraisal of included studies using the Newcastle–Ottawa Quality Assessment Scale for observational studies.

| **Study** | **Selection** | | | | | | **Comparability** | | **Outcome** | | | | | |
| --- | --- | --- | --- | --- | --- | --- | --- | --- | --- | --- | --- | --- | --- | --- |
|  | 1 | | 2 | 3 | 4 | | Cohort study | | 5 | | 6 | | 7 | |
| Kim et al,^1^ 2008 | | ☆ | - | ☆ | | ☆ | | ☆☆ | | ☆ | | ☆ | | ☆ |
| Castor et al,^2^ 2012 | | ☆ | ☆ | ☆ | | ☆ | | ☆☆ | | ☆ | | ☆ | | ☆ |
| Yanik et al,^3^ 2012 | | ☆ | ☆ | ☆ | | ☆ | | ☆☆ | | ☆ | | ☆ | | ☆ |
| Dai et al,^4^ 2014 | | ☆ | ☆ | ☆ | | - | | ☆☆ | | ☆ | | ☆ | | ☆ |
| Ambrosioni et al,^5^ 2015 | | ☆ | ☆ | ☆ | | ☆ | | ☆☆ | | ☆ | | ☆ | | - |
| Ananworanich et al,^6^ 2015 | | ☆ | ☆ | ☆ | | - | | ☆☆ | | ☆ | | ☆ | | - |
| Zhao et al,^7^ 2015 | | ☆ | ☆ | ☆ | | ☆ | | ☆☆ | | ☆ | | ☆ | | ☆ |
| Stekler et al,^8^ 2015 | | ☆ | ☆ | ☆ | | ☆ | | ☆☆ | | ☆ | | ☆ | | ☆ |
| Colby et al,^9^ 2016 | | ☆ | ☆ | ☆ | | - | | ☆☆ | | ☆ | | ☆ | | ☆ |
| Panichsillapakit et al,^10^ 2016 | | ☆ | ☆ | ☆ | | ☆ | | ☆☆ | | ☆ | | ☆ | | ☆ |
| Rutstein et al,^11^ 2019 | | ☆ | - | ☆ | | ☆ | | ☆☆ | | ☆ | | ☆ | | ☆ |
| Visseaux et al,^12^2019 | | ☆ | ☆ | ☆ | | ☆ | | ☆☆ | | ☆ | | ☆ | | ☆ |

Note: 1. Representativeness of the exposed cohort; 2. Selection of the non-exposed cohort; 3. Ascertainment of exposure to implants; 4. Demonstration that outcome of interest was not present at start of study; 5. Assessment of outcome; 6. Follow-up was long enough for outcomes to occur; 7. Adequacy of follow up of cohorts.

**References：**

1. Kim CO, Chin BS, Han SH, et al. Low prevalence of drug-resistant HIV-1 in patients newly diagnosed with early stage of HIV infection in Korea. *Tohoku J Exp Med.* 2008;216(3):259-265.

2. Castor D, Low A, Evering T, et al. Transmitted drug resistance and phylogenetic relationships among acute and early HIV-1-infected individuals in New York City. *Journal of Acquired Immune Deficiency Syndromes.* 2012;61(1):1-8.

3. Yanik EL, Napravnik S, Hurt CB, et al. Prevalence of transmitted antiretroviral drug resistance differs between acutely and chronically HIV-infected patients. *Journal of acquired immune deficiency syndromes (1999).* 2012;61(2):258-262.

4. Dai L, Li N, Wei F, et al. Transmitted antiretroviral drug resistance in the men who have sex with men HIV patient cohort, Beijing, China, 2008-2011. *Viral Immunol.* 2014;27(8):392-397.

5. Ambrosioni J, Sued O, Nicolas D, et al. Trends in Transmission of Drug Resistance and Prevalence of Non-B Subtypes in Patients with Acute or Recent HIV-1 Infection in Barcelona in the Last 16 Years (1997-2012). *PLoS One.* 2015;10(6):e0125837.

6. Ananworanich J, Sirivichayakul S, Pinyakorn S, et al. High prevalence of transmitted drug resistance in acute HIV-infected Thai men who have sex with men. *Journal of acquired immune deficiency syndromes (1999).* 2015;68(4):481-485.

7. Zhao B, Han XX, Xu JJ, et al. Increase of RT-Related Transmitted Drug Resistance in Non-CRF01_AE Among HIV Type 1-Infected Men Who Have Sex With Men in the 7 Cities of China. *Jaids-Journal of Acquired Immune Deficiency Syndromes.* 2015;68(3):250-255.

8. Stekler JD, McKernan J, Milne R, et al. Lack of resistance to integrase inhibitors among antiretroviral-naive subjects with primary HIV-1 infection, 2007-2013. *Antiviral therapy.* 2015;20(1):77-80.

9. Colby DJ, Crowell TA, Sirivichayakul S, et al. Declining trend in transmitted drug resistance detected in a prospective cohort study of acute HIV infection in Bangkok, Thailand. *Journal of the International AIDS Society.* 2016;19(1):20966-20966.

10. Panichsillapakit T, Smith DM, Wertheim JO, Richman DD, Little SJ, Mehta SR. Prevalence of Transmitted HIV Drug Resistance Among Recently Infected Persons in San Diego, CA 1996-2013. *Journal of acquired immune deficiency syndromes (1999).* 2016;71(2):228-236.

11. Rutstein SE, Chen JS, Nelson JAE, Phiri S, Miller WC, Hosseinipour MC. High rates of transmitted NNRTI resistance among persons with acute HIV infection in Malawi: implications for first-line dolutegravir scale-up. *AIDS Res Ther.* 2019;16(1):5-5.

12. Visseaux B, Assoumou L, Mahjoub N, et al. Surveillance of HIV-1 primary infections in France from 2014 to 2016: toward stable resistance, but higher diversity, clustering and virulence? *J Antimicrob Chemother.* 2019.
